# Contagious Caprine Pleuropneumonia: Seroprevalence and Risk Factors in Goats in Derashe Zone, Southern Ethiopia

**DOI:** 10.1002/iid3.70257

**Published:** 2025-09-18

**Authors:** Minale Getachew, Ephrem Tora, Nejib Mohammed, Teferi Benti

**Affiliations:** ^1^ Arba Minch University Arba Minch South Ethiopia Ethiopia; ^2^ Animal Health Institute Sebeta Ethiopia

**Keywords:** contagious caprine pleuropneumonia, Derashe, flock, goats, mixed effect logistic regression, seroprevalence

## Abstract

**Background and Objectives:**

*Mycoplasma capricolum* subspecies *capripneumoniae* is the causative agent of contagious caprine pleuropneumonia (CCPP), a highly infectious and economically significant disease affecting goats and sheep. CCPP is characterized by high morbidity and mortality rates, leading to substantial financial losses in affected regions. The aim of this study was to estimate seroprevalence and pinpoint risk factors for the occurrence of CCPP in the study area.

**Materials and Methods:**

A cross‐sectional study employing a multistage cluster sampling technique was carried out in the Derashe zone in 2021. In the study area, 426 goats from six villages and three clusters were tested for CCPP serostatus using the Competitive Enzyme‐Linked Immunosorbent Assay (c‐ELISA). Goat level CCPP was predicted by a mixed‐effect logistic regression model, while flock level CCPP was tested for association with risk factors using Chi‐square test.

**Results:**

Serum sample results revealed a 4.7% seroprevalence at the goat level (95% CI: 2.9–7.6), and a 52% prevalence at the flock level (95% CI: 31.3–72.2). The presence of health problems were associated with 4.2 times greater odds of CCPP seropositivity than those without health problems (OR = 4.2; *p* = 0.000). Goats found in large flock sizes were 5.7 times more likely to suffer a CCPP seropositivity than small flock sized goats owned together (OR = 5.7; *p* = 0.009).

**Conclusion:**

Given that the Derashe zone serves as a key livestock movement corridor with goats frequently transported to central regions of Ethiopia, and that contagious caprine pleuropneumonia demonstrated a high flock‐level prevalence across all study villages and clusters, it is crucial to implement a routine vaccination program to effectively control the disease.

**Abbreviations**
CCPP: contagious caprine pleuropneumonia
CI: confidence interval
OIE: Office International des Epizooties
$x^{2}$
: Chi‐squared test

## 1 Introduction

Ethiopia has over 50.5 million goats, which make up one of the most economically important livestock species [1]. Since goats have good adaptability, short generation intervals and a high rate of multiple births, large proportions of farmers depend on them for their livelihoods [2, 3]. While goats are known for their ability to adapt to harsh environments, the cumulative effects of overcrowding, poor nutrition, and diseases could have a serious adverse effect on their productivity [4]. Among the diseases, contagious caprine pleuropneumonia (CCPP) which severely affects goat production is a classical, acute disease causing major economic losses [5].

CCPP in goats is a life‐threatening disease with a high morbidity and mortality rate [6]. Despite the disease being limited to goats, there have been reported cases of subclinical infection in sheep and some wild ruminant species [7, 8]. CCPP is caused by a strain of *Mycoplasma capricolum* subspp *capripneumoniae*, originally known as the F38 biotype [9]. The first time this organism was isolated and shown to cause CCPP was in Kenya. Mccp belongs to the *M. mycoides* cluster, a group of six serologically and genetically related organisms namely: *Mycoplasma mycoides* subspp., mycoides Large colony (MmmLc), *Mycoplasma mycoides* subspp. mycoides Small colony (MmmSc), *Mycoplasm mycoides* subspp. capri, (Mmc), *Mycoplasma capricolum* subspp. Capricolum (Mcc), *Mycoplasma capricolum* subspp. c*apripneumoniae* and Mycoplasma group 7 strains [10, 11].

CCPP is a serious WOAH (World Organization for Animal Health)‐listed disease affecting goats that involves various clinical signs [12]. When CCPP affects the respiratory system, it manifests anorexia, fever, and respiratory signs like dyspnea, polypnea, productive coughing, grunting, inability to move, standing with their front legs wide and apart, stiff and extended necks, and constant salivation [13, 14]. Upon postmortem examination, fibrinous pleuropneumonia is found, along with massive lung hepatization and pleurisy, accompanied by the accumulation of straw‐colored pleural fluid [15, 16]. Reported morbidity and mortality rates may reach up to 90% and 60%, respectively, particularly during outbreaks in immunologically naïve goat populations [12].

Different factors may contribute to the spread of CCPP from one territory to another. Several risk factors contribute to its spread, including high‐density farming, where close contact between animals facilitates transmission. Poor ventilation in shelters increases the buildup of infectious droplets, while stressful conditions—such as transportation, malnutrition, or extreme weather—weaken immunity, making goats more susceptible [6, 17]. Lack of vaccination in endemic regions significantly raises outbreak risks, and introduction of infected animals into herds without proper quarantine further spreads the disease [18]. Moreover, co‐infections with other respiratory pathogens can worsen the severity of CCPP [8, 16]. Co‐infectious agents include bacteria (*Pasteurella*, *Chlamydia*, *Bordetella*, and *Mycoplasma*), viruses (*Parainfluenza* 3, *Morbilivirus*, and *Reovirus*), and fungi [16]. According to reports, *Mahnemia haemolytica* is the most common bacteria associated with respiratory infections in goats [19]. Effective control requires improved herd management, biosecurity measures, and vaccination programs.

CCPP has long been hypothesized to exist in Ethiopia on the basis of clinical manifestations, post‐mortem lesions, and epidemiological results [15]. In 1990, it was finally confirmed to present in Ethiopia by isolation of Mccp from pleural fluid samples [15, 20]. The confirmation of CCPP presence, coupled with its high mortality rates, reduced milk and meat production, increased expenses for diagnosis, treatment, and control, as well as trade restrictions on goats and their products, collectively contribute to significant economic losses. So far, investigations have been done in Ethiopia that show the disease occurs in six regions. CCPP, however, was primarily recorded in the Ethiopia′s pastoral and agro‐pastoral regions, which are well‐known for their goat farming. In the study area, data on the prevalence, risk factors, and distribution of CCPP remain limited. On the other hand, conducting CCPP research is essential to produce evidence for policymakers to establish efficient CCPP′s preventive and management measures that maximize the goat population′s potential contribution to the region's and country's economy. Thus, this study was aimed to estimate the sero‐prevalence, to identify and quantify individual level risk factors that are associated with the occurrence of the disease in goats of the Derashe zone, Southern Ethiopia.

## 2 Materials and Methods

### 2.1 Study Area Description

Dirashe Zone is one of the zones in the South Ethiopia Regional State of Ethiopia. In 2011, the Segen Area Peoples Zone was established, which includes Dirashe woreda and the 3 former special woredas surrounding it. Geographically, Derashe zone is located at 5°35′24.4″N and 37°16′16.5″E. Derashe has 10 kebeles (local administrative unit or subunit within the Woreda) with diferent ethnic groups [1, 21]. The climate of the Derashe zone is classified into three regions: highland (38.89%), midland (16.67%), and lowland (44.44%). The mean annual temperature ranges from 15.1°C to 27.5°C, while the average annual rainfall varies between 600 mm and 1600 mm. In Derashe, livestock production system is characterized by minimal management input in terms of breeding management, disease control, and nutrition and feeding systems, which are mostly traditional and subsistence oriented The livestock population of the Derashe zone comprised of 148,902 cattle, 54,071 sheep, 94,722 goats, 25,379 donkeys, 62 horses, 139 mules and 199,362 poultry [22].

### 2.2 Study Animal

The study included 426 goats kept under traditional extensive farming methods. Samples were taken from goats that had never been vaccinated against CCPP, were more than 6 months old, and belonged to both sexes. Based on owner information and dental eruption, the age of the study goats was determined. According to the classification [23], young goats are between 1 and 1.5 years of age with up to four permanent teeth, and adults are over 1.5 years old with more than four permanent teeth.

### 2.3 Study Design, Sampling Technique and Sample Size

A cross‐sectional study was conducted during 2021 to estimate the sero‐prevalence and to assess the related risk factors for CCPP in goats in three clusters/villages of Derashe zone, Southern Ethiopia. A cluster is defined as a geographical area comprising more than five kebeles organized for administrative purposes. A kebele is the smallest administrative unit in Ethiopia, typically comprising over 500 households. The Derashe Zone Agricultural Office supplied official records of clusters, while designated representatives provided listings of associated villages. A multistage sampling technique was employed to select the study zones and villages. Initially, the Derashe Zone was selected using purposive sampling. Within the zone, three clusters were selected based on goat population density, and from each cluster, three villages were randomly chosen. In the second stage, villages were selected randomly from the compiled list to enhance geographical and demographic representativeness. At the third stage, herds were randomly selected from goat populations stratified by location. Finally, individual goats from these herds were selected using a two‐stage cluster sampling method. Accordingly, the cluster sample size determination formula was used based on the [24]. The sample size required was calculated using an expected 25.7% pooled seroprevalence of CCPP in the Ethiopia [5]. In each of the 25 flocks, a mean of 17 individuals of goats, were randomly sampled totaling 426 serum samples. The fitting formula of two‐stage cluster sampling for a 95% confidence interval is:

$$\text{Ts}\;=\;\frac{1.96^{2}g\left\{\text{Pexp}\left(1-\text{Pexp}\right)-Vc\right\}}{{\text{gd}}^{2}-1.96^{2}\text{Vc}}$$

where:

*Ts* = required sample size, *g* = number of clusters to be sample = 25, Pexp = expected prevalence, *d* = absolute precision, *Vc* = between‐cluster variance. The sample size was calculated assuming a Pexp = 25.7%, with *d* = ±5%, and *Vc* = 0.0158 (estimated from a previous CCPP study carried out in Ethiopia in 2010 [25]).

### 2.4 Data Collection Procedure

A sterile disposable needle and plain vacutainer tubes were used to collect 6 ml of blood samples from the jugular vein of each animal with a history of non‐vaccination. The blood samples were held overnight at 45°C, and then the sera were decanted into cryovials. The sera were allowed to stay in the refrigerator at −20°C in the microbiology laboratory of Arba Minch University. Lastly, the sera were transported to the Animal Health Institute (AHI), Sebeta, Ethiopia, in ice packs and stored at −20°C until they were analyzed.

At the time of blood collection, observation and owner interviews were also conducted regarding putative risk factors. In addition, individual goats were assessed for factors such as health problems, breed, age, sex, and other animal‐level factors. Individual goats' ages were estimated by dentation, and they were divided into two age groups: young and adult. The next variables were grouped as flock level determinants: open/closed contact with other animals; sheep in the flock; goat/sheep trade route in the area; presence of frequent mixing; flock size; husbandry system; and barn type. Flock sizes were categorized as small flock size (˂ 50 goats), medium large flock size (≥ 50 goats). Frequent mixing was expressed as coming into contact (mingling) with other animals over time (date to date).

### 2.5 Serological Examination

The IDEXX CCPP competitive enzyme‐linked immunosorbent assay (c‐ELISA) was used to test the collected serum samples. In accordance with the manufacturer′s instructions, antibodies against Mccp were serologically detected using a commercial c‐ELISA kit (IDEXX, Montpellier, France). Using an ELISA microplate reader at 450 nm, optical density (OD) was measured. As a result, the following formula was used to interpret the results: percent of inhibition (PI) = (OD monoclonal antibodies, Mab − test serum)/(OD Mab − OD conjugate) × 100. The results are considered positive when PI% is greater than or equal to 55%.

### 2.6 Data Analysis

First data was entered, formatted, coded, and cleaned in Microsoft Excel, and then R Studio 4.1.3 was used to visualize, summarize and inference analysis of data [26]. Using descriptive statistics, such as frequency and prevalence, the study population′s characteristics and the proportion of CCPP across explanatory variable categories were described. A multilevel mixed‐effect logistic regression model was used to estimate the parameters. This model includes cluster‐specific random effects that allow the CCPP serostatus correlation between and within clusters to be accounted for. Village was considered a random influence in this study. Lack of independence between levels of nested data (individual goats nested within a flock, flocks within villages) was used to be explained by the model. Univariate mixed effect logistic regression was utilized to determine risk factors connected to CCPP serostatus at the individual level. The association of potential risk factors for flock‐level variables was computed using the Chi‐square test. For between‐herd data analysis, we used 25 independent clusters, as there is no sharing between flocks. Given the small sample size and the assumption of herd independence, the Chi‐square test is considered appropriate. Statistical association was cut at 5% (*p* < 0.05).

## 3 Results

### 3.1 Seroprevalence of CCPP

A sera sample of 426 goats was collected from 47 flocks in 9 villages and 3 clusters. Goats tested seropositive for CCPP were found in every village. While the flock level prevalence was 13/25 (52%) (95% CI: 31.3–72.2), the total goat level seroprevalence was 4.7% (95% CI: 2.9–71.6). The highest goat level CCPP prevalence was observed in Holite cluster in the zone followed by Gato cluster; while, the lowest animal level prevalence was observed in Walayite (Table 1).

**Table 1:** Goat and flock level CCPP seroprevalence by the Derashe zone in Southern Ethiopia.

Clusters	Villages	No. of flocks sampled	No. of positive flocks	Flock level prevalence (95% CI)	No. of goats sampled	No. of positive goats	Goat‐level prevalence (95% CI)
Gato	Village 1	3	1/3	33.3[0.8 – 90.6]	44	2	4.5[0.5–15.4]
	Village 2	3	2/3	66.7[9.4 – 99.1]	47	1	2.2[0.1–4.5]
	Village 3	2	1/2	50.0[1.3 – 98.7]	44	3	6.8[1.4–18.6]
Holite	Village 4	3	3/3	100[29.2 – 1]	34	6	17[6.7–34.5]
	Village 5	3	1/3	33.3[0.8 – 90.6]	52	2	3.8[0.4–12.3]
	Village 6	3	1/3	33.3[0.8 – 90.6]	46	1	2.1[0.1–11.2]
Walayite	Village 7	2	1/2	50.0[1.3 – 98.7]	46	1	2.1[0.1–11.2]
	Village 8	3	0/3	—	47	0	—
	Village 9	3	3/3	100[29.2 – 1]	46	4	8.7[2.4–20.8]
Total		25	13	52[31.3 – 72.2]	426	20	4.7[2.9–7.6]

### 3.2 Association of Individual Goat Level Risk Factors With CCPP Occurrence

Potential risk factors such as age of goats, and presence of health problem, flock size, body condition, accessibility to water and sex of goat were considered for the goat level risk factor analysis. The results of univariable mixed‐effect logistic regression model are presented in Table 2. In this analysis, the presence of health problems, flock size, and body condition score were statistically significant factors.

**Table 2:** Goat level univariable analysis of potential risk factors for CCPP serostatus using mixed effect logistic regression model taking cluster, village and flock as random effect.

Variable	Category	Number of animals examined	Number positive	Proportion of seroreactors in (95% CI)	Crude odds ratio (95% CI)	*p* values
Age	Young	55	5	9.3[3.5–16.7]	Ref.	
	Adult	355	15	4.2[2.5–6.7]	2.2[0.6–6.5]	0.144
Health problem	No	314	8	2.5[1.2–4.9]	Ref.	
	Yes	112	12	10.7[5.6–17.9]	4.2[1.5–12.2]	0.000
Flock size	Small	165	2	1.2[0.14–4.3]	Ref.	
	Large	261	18	6.9[4.2–10.6]	5.7[1.3–51.0]	0.009
Body condition score	Good	180	3	1.7[0.3–4.7]	Ref.	
	Moderate	156	4	2.5[0.7–6.4]	1.5[0.2–10.6]	0.573
	Poor	90	13	14.4[7.6–23.4]	8.6[2.3–48.2]	0.000
Sex of goat	Male	113	9	7.9[3.9–13.6]	Ref.	
	Female	293	11	3.7[2.2–6.4]	2.2[0.7–5.7]	0.097
Accessibility to watering system	Assessable	386	19	4.9[2.9–7.5]	Ref.	
	Moderate	20	1	5.0[0.2–2.4]	1.2[0.02–7.1]	0.998

### 3.3 Association of Flock Level Risk Factors with CCPP Occurrence

A chi‐square test was used to determine the independent association between the proportion of CCPP occurrences and risk factors at the flock level as shown below (Table 3). Based on this test, risk factors such as flock size, trade route and sheep presence in the flock were statistically significantly associated.

**Table 3:** The chi‐square association of flock level (*n* = 25) risk factors with CCPP occurrence.

Variable	Categories	Outcome		Test for independence	
		Frequency	Positive	Chi‐square	*p* value
House type	Fenced stable	6	3		
	Housed barn	12	11	4.54	0.1034
	Both type	7	6		
Husbandry system	Extensive	16	15	3.14	0.0765
	Pastoral	9	5		
Flock size	Large (≥ 50)	14	14	5.37	0.0205
	Small (< 50)	11	6		
Frequent mixing	Mingling	17	15	0.93	0.3347
	Over time	8	5		
Trade route	Yes	18	18	9.76	0.0017
	No	7	2		
Contact with other animals	Yes	15	14	2.34	0.1258
	No	10	6		
Sheep presence in flock	Yes	14	14	5.37	0.0205
	No	11	6		

## 4 Discussion

The current study area, Derashe zone, has a trade route where goats are raised and marketed. In Derashe zone, due to the nature of the trade route, goats are transported frequently, so that contagious diseases are likely increasing the risk of disease transmission. Among the goat systems of production in Derashe, Southern Ethiopia, CCPP has an overall prevalence of 4.7% [2.9–7.6] and 52% [31.3–72.2] at the individual goat and flock levels, respectively, thereby making it a significant disease. Considering that CCPP is contagious, and individual cases can result in herd‐level transmission, the current study results suggest strong biosecurity measures, preferably vaccination, and enhanced strategies for control and eradication of CCPP, including test and slaughter. This procedure could also be complemented by additional measures to minimize transmission risk (e.g., in enzootic regions, restrictions on animal movement; testing and culling of infected animals). During an outbreak, stamping‐out procedures are applied) [27].

CCPP, as documented, is a highly transmissible and economically significant disease impacting goat populations across more than 40 countries, with the highest prevalence in Africa, Asia, and the Middle East [6, 19, 28, 29, 30]. The potential spread of CCPP to other continents like European union and America is poorly documented, but it could theoretically occur through animal imports from endemic regions [29, 31]. MoLAF [32] states that CCPP is a major animal health concern for goats in Ethiopia, with reports of outbreaks coming from practically every part of the country. CCPP seroprevalence among goats and associated risk factors in the Derashe zone have never been studied before. Therefore, the findings of this study contribute substantially to our understanding of CCPP dynamics in the Derashe zone of southern Ethiopia and its epidemiology as well as to the development of effective prevention strategies.

In this study, the goat level seroprevalence of CCPP was 4.7%, which is in line with a study conducted by [15] who reported 5% from the Amhara region, Ethiopia. The reported seroprevalence was lower than that reported by [25] and [33], where they documented prevalence of 18.6% and 31.2%, respectively. The current finding also much lower than Ethiopian National level pooled estimated seroprevalence of 25.7% reported by [5] using systematic review and meta‐analysis. Several other studies have documented prevalence levels that are higher than current finding by Kipronoh et al. [34] reported a prevalence 47.2% in the Rift Valley region of Kenya; Ingle et al. [35] reported a prevalence 33.67% in the Nagpur district Vidarbha region of Pakistan, and Mbyuzi et al. [36] reported a prevalence 52.1% in the southern zone of Tanzania. In contrast to the present study, differences in animal husbandry, sampling strategy, sample size, agro‐ecology, population density, time of sampling, laboratory test used, and study area could explain the variation in estimated seroprevalence [37, 38].

In this study, relatively higher 7.6% goat level prevalence was observed in Holite cluster of the Derashe zone. Compared to other clusters, this is likely that it is due to free animal movement within the cluster, trade routes within the village, and communal grazing practiced with the same water source. A susceptible animal can be in contact with an infected animal under these conditions. Gumayde and Konso zone are known for the transit of live animals and are therefore regarded as endemic and foci of CCPP for this zone's clusters. In addition, animal movement occurs in these areas from Gumayde to Holite cluster and from Konso zone to Gato cluster through informal trade and markets. Since its contagious nature, CCPP can spread to other animals when there is a freely movement of animals [12].

A seroprevalence of around 52% was observed at flock level in the present study. It was considered positive if at least one goat was seropositive in the flock. The current report is comparable to Mekuria and Asmare [25] who reported 50.5% prevalence in southern Ethiopia, and Kipronoh et al. [34] reported 54.83% in Kenya. However, this flock level prevalence is higher than the 9.6% reported in Tanzania Swai et al [39]. However, proportions of seroprevalence in lowland, Hammar and Bena Tsemay districts, were higher at 73.3% and 62.1%, respectively both pastoral areas in southern Ethiopia [25]. Likewise, prevalence studies performed across the enzootic range in Kenya showed that all herds presented for CCPP vaccination were tested positive [40]. In this study, relatively comparable flock level CCPP seropositivity observed compared to others could be a signal of new animal entry to the flock for breeding and flock expansion purposes.

Among animal level risk factors, presence of health problems, flock size, and body condition score were found statistically significant (*p *˂ 0.05). Goats reared in large herd sizes were 5.7 times more likely to be seropositive than those in small herds. This report is in agreement with the report of Bekele [41], Regassa et al. [42], Parray et al. [43] and Selim et al. [44] who reported a significant association between CCPP seropositivity and herd size. In accordance with some authors reported a positive correlation between CCPP seropositivity and herd size increment in endemic areas. The possible explanation is that, as the number of infected and uninfected animals mix in larger flocks, CCPP prevalence is higher in larger flocks due to a higher persistence of Mccp in different animals [34, 43]. As a result of direct contact, CCPP is mainly transmitted by aerosols, droplets, and nasal discharge [6]. Because intensive management in large flocks is difficult compared to small flocks, pastoralism of large flocks allows animals from different flocks to mix, spreading the infection.

When goats exhibiting clinical signs were compared to clinically healthy goats, the odds of testing positive for CCPP were significantly higher among those with clinical disorders (OR = 4.2, *p* = 0.000). This finding aligns with the results of Lugonzo et al. [45], who reported that animals' immune systems were weakened as a result of stress from other infections, ultimately making more susceptible to respiratory illnesses like CCPP. It has also been shown that CCPP severity may be exacerbated by coexisting viral infections, potentially other mycoplasma infections, stress, and unfavorable climatic conditions [12, 17]. Concurrent infections with two or more pathogens are a common cause of health problems, and interactions between numerous pathogens frequently seem to produce a more severe consequence than with an individual pathogen [46, 47].

This study found that sheep and goats kept together were 100% seropositive (Table 3). Therefore, sheep presence has a statistically significant association with CCPP seropositivity in goat flocks ($x^{2}$ = 5.37; *p* = 0.0205), provided that sheep in contact with infected goats were seropositive. In agreement with our observation, previous authors reported that sheep were seropositive from different parts of Ethiopia [15, 33, 48, 49]. For instance, according to Hadush et al [48] 47.6% of sheep were found sero‐positive in the Tigray region, northern Ethiopia. Mbyuzi et al. [36] reported a seroprevalence of 36.7% from sheep serum in Tanzania. Moreover, Mcpp has been isolated from sheep with respiratory diseases [50], from healthy sheep that have come into contact with CCPP‐affected goat herds [7], from sick sheep mixed with goats [13], as well as from sheep′s lung and nasal swabs by Cetinkaya et al. [51]. This raises a debate as sheep play a critical role in maintaining *Mycoplasma capricolum* subspp *capripneumoniae* transmission by acting as reservoirs. According to this, sheep might contribute to the epidemiology of the disease by causing goats to become infected. Since mixed herding of both species is very common in Ethiopia and other parts of the world, it seems relevant and worth investigating further.

The results of this study revealed that 4.7% of individuals and 52% of herds were infected with caprine pleuro‐pneumonia. The herd level prevalence is too high since CCPP is a contagious disease, and a single case occurring in a given farm may disseminate the disease to other surrounding herds. The study procedure was only a serological survey, and the study design was a cross‐sectional study. The study design has limitations that could affect the inference to an external population because the cause‐effect relationship following the cross‐sectional study design was weak. It is common for serological tests to use natural proteins as the defining antibody‐capturing reagent, which limits the specificity and sensitivity of a serodiagnosis. It has also important to note the molecular epidemiology in understanding CCPP strain diversity. However, due to logistical constraints, lack of infrastructure, and funding limitations, our study prioritized serological aspects. While this does not explore genetic, variations, our data provide a foundation for future molecular studies by identifying high‐risk areas and populations where such analyses could be targeted.

## 5 Conclusion

This study confirms the presence of CCPP in goats in the Derashe zone in southern Ethiopia, which is a serious and economically important disease of goats. The animal level seroprevalence of CCPP in goats is low, while flock level seroprevalence is higher. This study found that some factors at the goat and flock levels were significantly associated with CCPP seropositivity. Body condition score status, flock size, and presence of health problems were found to be significantly associated with CCPP seropositivity at the animal level, while presence of sheep, flock size and presence of trade routes were found to be significantly associated at the flock level. At flock level single CCPP case presence can reveal that the infection persists within the flock and can spread to other noninfected flocks or areas. Therefore, it is necessary to develop suitable preventive and therapeutic methods for the treatment and management of CCPP in and around this trade route region. As such, to enhance the productivity of goat industry, sufficient and well‐coordinated measures must be implemented to effectively control the disease.

## Author Contributions

**Minale Getachew:** conceptualization, data curation, formal analysis, investigation, methodology. **Ephrem Tora:** conceptualization, data curation, formal analysis, investigation, methodology, project administration, software, supervision, validation, writing – original draft, writing – review and editing. **Nejib Mohammed:** conceptualization, data curation, investigation. **Teferi Benti:** data curation, formal analysis, investigation, methodology.

## Ethics Statement

Arba Minch University Research Ethics Review Committee has approved this study before it is carried out. Verbal consent was obtained for the sera sample collection and animal owners were informed that the results of the samples would be used solely for research purposes.

## Consent

The authors have nothing to report.

## Conflicts of Interest

The authors declare no conflicts of interest.

## Data Availability

The datasets supporting the conclusion of this article are included within the article.
